# Olive oil, Greek Mediterranean diet heritage and honoring the past to secure our future: Priorities for research and education

**DOI:** 10.3389/fnut.2022.1058402

**Published:** 2022-11-23

**Authors:** Antonia Trichopoulou

**Affiliations:** ^1^Hellenic Health Foundation, Athens, Greece; ^2^Academy of Athens, Athens, Greece

**Keywords:** Mediterranean diet, public health nutrition, national survey, dietary patterns, modern consumer, traditional foods, dietary supplements, sustainability

## Introduction

The Nobel prize winning Greek poet Odysseas Elytis wrote, “*If Greece is completely destroyed, what will remain is an olive tree, a vine and a boat; this is enough to begin again”* (1). Indeed, some plants, like the olive tree, and the grapevines, have evidently been in Greece forever. In the 1953 Rockefeller Report entitled “Crete: a case study of an underdeveloped area,” Allbaugh mentions: “*olives, cereal grains, pulses, fruit, wild greens and herbs, together with limited quantities of goat meat and milk, game, and fish consist the basic Cretan foods... no meal was complete without bread… Olives and olive oil contributed heavily to the energy intake... food seemed literally to be “swimming” in oil”* (2). In traditional Greek cuisine, olive oil is used in almost all culinary practices and applications. The cooking term *ladera*, originates from the word *ladi*, Greek for oil, and generally describes vegetables cooked in plenty of olive oil, onions, garlic, tomatoes and various herbs. These are the initial steps of making *ladera* dishes and the cooking practice of Greek cuisine called *tsigarisma* (sautéing)—basically to “sauté.” Onions and garlic would first be wilted or softened, for seconds, in a frying pan with a few tablespoons of hot olive oil, a practice that adds a certain depth of flavor. Subsequently, vegetables, grated tomatoes, various aromatic herbs, and a small amount of water is then added and the food is essentially left to cook on its own, traditionally over a low flame (3).

This combination of tomato, olive oil, garlic, onion and herbs in *ladera* increases the amount of polyphenols and carotenoids. Lightly frying vegetables with olive oil makes the vegetables healthier. Olive oil has the ability to act as a food excipient, which helps to release and absorb bioactive compounds from garlic, onion and tomato (4)^1^.

Indeed, we know the importance of olive oil both culturally and for health—but do we still respect our heritage? What are Greek people eating today? Are they still eating the “healthy” traditional Mediterranean diet (5)? A diet with olive oil at its core and which has been a central part of Greek culture and daily life evidently forever (6).

## Greek national health and nutrition survey -HYDRIA and dietary patterns

Answers to these questions are offered by the Greek National Health and Nutrition survey—HYDRIA, where food and macronutrient intake of the population in Greece were investigated along with an evaluation of adherence to the traditional Greek Mediterranean diet. In this survey, implemented in 2013–2014, adults over 18 years old (*n* = 4,011) were included (7). The HYDRIA survey design followed the recommendations of the European Health Examination Survey (EHES) (8) and the European Food Safety Authority (EFSA) (9) for the collection of health and dietary data at national level, allowing for inter-country comparisons (10).

The HYDRIA survey is a nationally representative survey. The selection of eligible individuals was based on the latest Greek general population census completed in 2011, of men and women aged 18 years and over, residing permanently in the country. The sampling frame covered all the 51 prefectures of the 13 regions of Greece and implemented a harmonized EU Member States protocol to collect data which allowed for direct comparison with other European countries (10). The HYDRIA survey provides a robust nationally representative sample, standardized data collection, and a comprehensive picture of today's Greek dietary pattern (7). Key elements of the findings provide important insights. Significantly, it was found that the contribution of total fat in adults was higher than the 20–35% of dietary reference intake ranges suggested by EFSA (11). In both men and women, 42–43% of total energy intake was from fat. Monounsaturated fatty acids (MUFA) represented 20.3% of men's and 20.6% of women's total energy intake. Saturated fatty acid (SFA) represented 13.1 and 13.5% of men's and women's total energy intake, respectively. Polyunsaturated fatty acid (PUFA) represented 5.7% of men's total energy intake with 5.9% for women. A higher MUFA percentage of total energy intake was noted for those individuals above 65 years of age at 21.9–21.3 vs. 20.1% for the younger adults surveyed. The high consumption of monounsaturated fats from olive oil was the main contributor of overall fat intake, with median distribution of the usual daily intake from fats and oils being 44 g for men and 32 g for women overall, with olive oil alone representing 34 g and 25 g for men and women, respectively (11).

When looking at the usual mean intakes in a diet of 2,000 kcal/day, it becomes clear that both men and women over the age of 65 eat less meat than their younger counterparts, balanced by more fish, dairy, legumes, fruit and vegetables (excluding potatoes), and olive oil. Similarly, older men and women have a higher median intake of fats and oils at 46 and 32 g/day, respectively, mainly from olive oil, than younger age groups. Younger adults eat more meat, cereals and sugar products, along with consuming more non-alcoholic and alcoholic beverages (11).

## Understanding dietary supplement use

The HYDRIA survey provides comprehensive data on dietary supplement use in Greece. Dietary supplements (DS) are used by 31% of the overall Greek population. DS use is higher by those living in urban areas and a considerably higher percentage of women, 40%, take DS in Greece, particularly those who eat a higher amount of fruit and those with a chronic medical condition. Men DS users represent only 22% of the population and tend to be those who have a good or very good self-reported health status (12).

Multivitamins with minerals (MVM) were reported most frequently at 5.4% of DS users, with men preferring this type of supplement. Following that, Iron at 4.6%, and Calcium DS are consumed by 4.7% of users and more often by women and survey participants with lower education levels. Plant- and oil-based supplements were used by <5% of the participants (12).

With studies finding that dietary supplements (DS), in many cases, are not being used to supplement nutrient deficiencies but on the understanding that they play a preventive role against disease (13), further exploration is warranted as to whether this “nutritional transition” (14) and its newly established habits actually benefit health. In addition, the efficiency of recommended food intake in comparison to substituting intake with DS is a pertinent question.

## Addressing the move away from traditional dietary patterns

There is clear evidence that a significant portion of the population of Greece has moved away from the “healthy” traditional Mediterranean diet, with olive oil at its core. The HYDRIA survey population representative sample shows that, overall, only 28.3% of adults are now characterized as having a high adherence (score of 6–9 points) to the Mediterranean diet. About 39.7% of participants over 65 years old and 25.5% of participants under 65 years old were included in the high adherence category of the Mediterranean diet score (11).

Key is the observation that adults consume levels of red meat, fruit and vegetables that are not in line with international dietary recommendations. Based on the criteria, it is clear international dietary recommendations are not being met by most adults when assessing their intake levels of several macronutrients and selected foods. With these insights into the dietary habits of adults in Greece it is possible to note that younger adults eat more meat, dairy and alcohol, which moves away from the lower to mid-level amounts that constitute the traditional Mediterranean diet consumption levels. They also eat lower amounts of fruit, legumes and vegetables, although older participants had comparably higher consumption in these categories (11). Even with contemporary moves toward acknowledging the benefits of the traditional Mediterranean diet translating into support for modifying dietary patterns toward incorporating key elements of the Mediterranean dietary pattern (15), younger generations in Greece are still moving away from beneficial food choices (11). The potential detrimental effects on mortality and morbidity in these observations are becoming clear (16).

Changes in diet could be attributed to the documented life-style changes impacted by the urbanization process. Urban living can have enormous and complex impacts on diets including increased participation in the workforce which has corresponded to moves toward convenience foods rather than quality food choices, increased income levels which enable access to a larger range of foods which are not necessarily as nutritious, the wider availability of inexpensive poor nutritional quality foods (frequently of animal origin), and more readily available unhealthy packaged foods (17).

The HYDRIA survey noted differences in adherence to the Mediterranean diet were observed among the four main geographical regions in Greece, in the highly urbanized area of Attica the Mediterranean dietary pattern was notably lacking with 35.6% of participants having a low level of adherence (11). Parallel with other studies the observed influence of urbanization and regional difference in diet quality emphasizes the need to consider tailored regional nutrition strategies (18).

The overall improvement in socioeconomic conditions in Europe and the globalization of the food supply has contributed to changes in amounts, types and costs of foods available across Europe and beyond. Just as policies should be distilled to provide regional effectiveness, attention should not be taken away from the effect of global economic policies such as those on trade, marketing and investment, and related global food and health policies have at all levels of stratified policy-making (19).

## Tradition rarely honors unhealthy habits

Although there are some exceptions, tradition rarely honors unhealthy habits (20) with the United Nations Educational, Scientific and Cultural Organization (UNESCO) enshrining the intangible heritage of the Mediterranean diet and defining it as “*… a set of skills, knowledge, rituals, symbols and traditions concerning crops, harvesting, fishing, animal husbandry, conservation, processing, cooking, and particularly the sharing and consumption of food… and a way of life guided by respect for diversity”* (20, 21).

In the past decades the pressures of modern life have changed the way people eat in Greece, resulting in spending less time sharing food and eating together. This loss of shared preparation and consumption of food generates a decline in the transfer of knowledge and skills related to food. In order to ensure the preservation of the traditional Mediterranean diet, before all that remains “*is an olive tree, a vine and a boat*” (1) priority could be the development of policies and actions related to the enhancement of knowledge and skills (e.g., “*tsigarisma”*-sautéing) related to food, at the very least in the populations that have a direct cultural heritage connection to it. Indeed, culinary habits could be a promoter of family and social cohesion at the local level. Education on the cultural elements inherent to the Mediterranean dietary pattern, extending healthy eating guidelines, through to training food producers, mass caterers, industry as well as faculty, students, and interested individuals, can work to preserve the traditional Mediterranean diet (22).

Considering the priorities for research and education on olive oil, as it is central to the Mediterranean diet, we assess each component of this dietary pattern, acknowledging that all are intertwined, since it is not easy to dissociate them. Within this context focus should be on the integrity and implementation of actions that will support and affect the development of compatible fiscal and pricing policies, school food and nutrition plans, food marketing, and nutrition labeling guiding principles (23).

One example is utilizing the current societal trend of “Healthy Eating” paralleled with an increased demand for traditional, local and seasonal foods, that can translate into business opportunities for the catering sector. The focus could be on traditional options emphasizing the use of olive oil and on the traditional dishes (24).

## Traditional foods

The nutritional value of the Greek Traditional Mediterranean diet could be attributed to the combination of its constituents and not to a single component (25). Culinary practices seem to play a crucial role in the bioactivity of their ingredients and beneficial effects. Cooking methods, for example, that involve soaking herbs in a warm liquid, such as in the process of making soup, increase the antioxidant capacity of the herb extract, while extracts taken after grilling had a lower antioxidant capacity compared to the uncooked herb extract. Steaming and sautéing have been reported to increase the antioxidant capacity and phenolic content of extracts taken after cooking (26).

Tomatoes, onions, garlic and aromatic herbs are principal elements of the culinary practices of the Greek diet. Herbs are rich in polyphenols, especially in their dried form, and generally contain higher amounts of polyphenols compared to other polyphenol-rich foods, such as dark chocolate, berries, and grapes. Polyphenols are well-known for their antioxidant properties and also for their anti-microbial, anti-diabetic Type II, and anti-asthma properties (27).

Accordingly, the systematic investigation of the nutritional value of traditional foods and recipes is needed, as well as the investigation of the historical and cultural identity of the simple and composite foods (recipes). In this way, scientific data would be gathered, which would substantiate the influence of the simple and composite foods on health.

Since in the preparation of traditional Mediterranean foods the use of olive oil is a central element, with its recognized health benefits, the study and rediscovery of these foods is crucial. Moreover, several traditional foods could represent healthy and ecologically friendly choices that also support local economies, because local products are generally used in their preparation. The cultivation of local products contributes to biodiversity and enhances the consumption of olive oil and to the employment of local people, thus promoting the balance between the territory and the people (28).

## Sustainable nutrition—honoring our past to secure our future

The traditional Mediterranean diet embodies a sustainable dietary pattern in which culture, wellbeing and the environment are required to interact in harmony. As a plant-based diet, characterized by the low intake of meat and meat products, emphasizing traditional foods with their seasonal and regional produce, it is recognized as a sustainable dietary pattern (28).

Yet when imbalances occur there is a risk of losing essential elements, for example, the threat to the biodiversity of categorized medical and aromatic plants (MAPs) in Greece that climate change and unregulated harvesting may pose (3). The current food system should be reviewed within the context of ensuring the preservation and innovation of products considered to be exemplary in a sustainable food system. Any reform has to involve local communities and consider local needs alongside national ones, still within a global context.

In keeping with the need for a return to traditional foods access to the traditionally available produce is key. Farmers' markets may be instrumental in finding ways to increase production and encourage people to increase their consumption, in particular vegetables, at the population-level, which are consumed not only as salads but as main dishes, in both cases with considerable use of olive oil. The UNESCO Mediterranean diet definition adds “*Markets also play a key role as spaces for cultivating and transmitting the Mediterranean diet during the daily practice of exchange, agreement and mutual respect*” (21). There are some indications that fruit and vegetable consumption was positively associated with the use of farmers' market shopping (29). Therefore, more information is needed regarding the use of farmers' markets, more specifically clarification of what are the barriers and facilitators to farmers' market shopping and the association between farmers' market access, use and socio-demographic characteristics of consumers.

The Mediterranean diet is more than just a “healthy diet,” it is essential to build on the now robust documentation of its health benefits to support the evidence of its role as a sustainable dietary pattern. The Mediterranean diet warrants the convergence of knowledge with further multi-disciplinary studies encompassing the key elements of this dietary pattern, including economic, sustainability, cultural and environmental dimensions (30).

## Mediterranean diet at the heart of a global approach to healthy dietary patterns

The respected contemporary report entitled 'Food in the Anthropocene: the EAT-Lancet Commission on healthy diets from sustainable food systems.', credits the Mediterranean diet as “*a diet that maximizes longevity, improves health-related quality of life and is ecologically sustainable and environmentally friendly.”* Significantly, the work details a universal healthy “reference diet” aimed at providing a way forward toward healthy dietary patterns as alternatives to standard current diets, many of which are high in unhealthy foods. This EAT “reference diet” encompasses many of the main elements of the traditional Mediterranean diet (15).

Practical approaches for dealing with any challenges in establishing the elements of the Mediterranean diet beyond the identified region of its heritage have already been tabled. Although issues still exist, examples such as the development of the Asian Diet Pyramid which respects the nutritional elements of the traditional Mediterranean Diet Pyramid and began over 30 years ago, following with the Latin American and African Heritage Diet Pyramids, represent efforts to transfer knowledge for the benefit of global populations (31). The concept of a “Planeterranean” diet is being addressed based on the premise that “*in every place of the world, it is possible to identify specific fruits, vegetables, legumes, wholegrain, and sources of unsaturated fats which present nutritional contents and characteristics similar to those provided by typical foods of Mediterranean diet, likely to have also similar health benefits for populations living far from the Mediterranean area”* (32). It is important, however, not to lose sight of the significance that the Mediterranean diet has at the global level, providing relevant characteristics of a sustainable diet beyond specific foods and nutrients (33).

## Discussion: Priorities for research and education

Upon investigation it is possible to conclude that respect still exists for the heritage of the healthy, traditional Mediterranean diet, yet adherence is low as lifestyle choices, policies and global systems are removing essential access to key elements.

There is a need to move from the evidence to policies based in a long-term plan in which evaluation and actions should include the development of and implementation of global, regional and local food and nutrition policy that takes into account not only health issues but considers economy, culture and the environment, with a focus on olive oil.

## Author contributions

The author confirms being the sole contributor of this work and has approved it for publication.

## Conflict of interest

The author declares that the research was conducted in the absence of any commercial or financial relationships that could be construed as a potential conflict of interest.

## Publisher's note

All claims expressed in this article are solely those of the authors and do not necessarily represent those of their affiliated organizations, or those of the publisher, the editors and the reviewers. Any product that may be evaluated in this article, or claim that may be made by its manufacturer, is not guaranteed or endorsed by the publisher.

## Footnotes

^1^On 3 May 2022, Dr Antonia Trichopoulou was invited to the Pontifical Academy of Sciences Conference on “The art of science of olive oil: nutrition, health and planetary health”, in Rome, and addressed the participants with the lecture entitled: “Olive Oil and the Heritage of the Greek Mediterranean diet: Priorities for Research and Education”. The address was based on the findings of the Greek National Health and Nutrition Survey HYDRIA.
